# The Transcriptional Regulator *TRG1* Plays a Role in Regulating Peptaibol Biosynthesis in *Trichoderma longibrachiatum*

**DOI:** 10.4014/jmb.2602.02013

**Published:** 2026-05-25

**Authors:** Weishe Hu, Aizhi Ren, Mengjiao Guan, Xiaoting Wang, Ming Li, Xiusheng Zhang, Peibao Zhao

**Affiliations:** 1 Liaocheng University, Liaocheng, Shandong 252000, P. R. China; 2 Shandong Xiehe University, Jinan, Shandong 250100, P. R. China

**Keywords:** *Trichoderma longibrachiatum*, Peptaibols, Non-ribosomal peptide synthetases (NRPSs), Transcription regulation

## Abstract

*Trichoderma* species are valued as potent biocontrol agents, largely attributable to their production of peptaibols, structurally diverse secondary metabolites known for antibacterial activity, induction of apoptosis, and enhancement of plant disease resistance. To better understand the regulatory mechanisms underlying peptaibol synthesis, this study characterized the transcription regulator gene *TRG1*, identified within a non-ribosomal peptide synthetase (NRPS) gene cluster in *T. longibrachiatum* SMF2. A Δ*TRG1* knockout mutant was successfully generated via homologous recombination. Phenotypic comparison with the wild-type strain revealed that the Δ*TRG1* mutant maintained comparable growth rate, colony morphology, and sporulation capacity. However, it showed significantly reduced antagonistic activity against the phytopathogen *Botrytis cinerea*, as well as diminished efficacy in controlling gray mold on detached rose leaves. High-performance liquid chromatography (HPLC) analysis provided direct evidence that peptaibol yield in the mutant decreased by approximately 2.5-fold relative to the wild type. Collectively, these results demonstrate that *TRG1* acts as a key positive transcriptional regulator essential for high-level peptaibol biosynthesis, likely through modulation of NRPS gene expression. Thus, *TRG1* represents a promising molecular target for bioengineering approaches aimed at improving the biocontrol performance of *Trichoderma* strains.

## Introduction

As global living standards rise, there is an intensifying focus on health, driving a growing interest in environmentally friendly agents for the prevention and control of crop diseases [1]. *Trichoderma* is the most commonly used biocontrol fungus, widely distributed in soil and plant rhizospheres [2, 3]. Its primary biocontrol mechanisms include competition, antibiosis, and the induction of host resistance [4, 5]. However, the widespread application of *Trichoderma* is limited by the inherent drawbacks of biopesticides, such as slow efficacy, narrow control spectrum, and susceptibility to environmental conditions [6, 7]. To overcome these limitations and expand its agricultural utility, a feasible strategy is to investigate the biocontrol mechanisms of *Trichoderma* at a molecular level, specifically by developing efficient biocontrol agents from its secondary metabolites.

*Trichoderma* produces various compounds, including peptaibols, which exhibit important functions such as antifungal activity, inducing cell apoptosis and enhancing plant disease resistance. These peptaibols are synthesized via non-ribosomal peptide synthetases (NRPSs) pathways [8-11]. In recent years, extensive research has elucidated the structure, function and biosynthetic pathway of NRPSs and this peptaibols [12-15]. Despite their molecular understanding, the broad agricultural and pharmaceutical application of NRPS-derived peptaibols faces significant challenges. NRPSs are the largest enzymes discovered to date, making artificial expression difficult. Furthermore, current genetic engineering techniques are typically limited to NRPSs that produce small peptides (3-5 amino acids) [13, 15, 16]. Therefore, strengthening the basic research related to the synthetic mechanism of peptaibols is crucial. Specifically, investigating the regulatory mechanisms of peptaibol synthesis at the transcriptional level and seeking to improve the peptaibol yield by enhancing NRPS expression through bioengineering are essential to unlock the full potential of *Trichoderma* and peptaibols in the control of plant diseases. Through bioinformatics analysis, more than ten NRPS-PKS gene clusters were identified in the genome of *T. longibrachiatum* SMF2. Previous studies have shown that Trichokonin VI, a 20-amino acid active peptide, is the most important active peptide produced by *T. longibrachiatum* SMF2, and its synthesis is governed by the NRPS gene *tlx1* [17, 18].

In this study, we aimed to identify and characterize a transcriptional regulator involved in peptaibol synthesis. We first used bioinformatics analysis to identify a transcriptional regulator (*TRG1*) within an NRPS gene cluster in *T. longibrachiatum* SMF2. We then created gene knockout mutants and demonstrated that the antifungal activity and production of peptaibols in the Δ*TRG1* mutant were significantly reduced. These findings strongly indicate that *TRG1* is a transcriptional regulator of peptaibol synthesis in *T. longibrachiatum* SMF2 and establishes a new target for the bioengineering-driven improvement of biocontrol agent production.

## Materials and Methods

### Fungi

The wild type strain *Trichoderma longibrachiatum* SMF2 was obtained from the Key Laboratory of Microbial Technology at Shandong University. The plant pathogen, *Botrytis cinerea* T_4_ was obtained from Prof. Dickman Marty of Texas A&M University. Both *T. longibrachiatum* SMF2 and *B. cinerea* T_4_ were routinely cultured on potato dextrose agar (PDA) or potato dextrose broth (PDB) medium at 25 ± 2°C [11]. Mycelia were collected and genomic DNA was extracted using a fungal genomic DNA kit (Invitrogen) for use as a template in subsequent PCR amplification and for molecular validation experiments.

### Bioinformatics Analysis of NRPS Cluster

Bioinformatics analysis of the genomic sequence surrounding the NRPS gene *tlx1* was conducted to identify the complete NRPS cluster and associated genes. The coding sequences and introns within the cluster were identified using the software DNAman and MEGA 11. The Promoter and homologous genes were analyzed using online software promoter 2.0 and BLASTn to infer the functions of the identified genes, particularly potential regulators (like transcription factors) or tailoring enzymes involved in peptaibol synthesis.

### Construction of Knockout Vectors of the Gene *TRG1* and Mutant Screening

Based on the principle of homologous recombination, the *TRG1* gene-disruption vector pUCATPH-*TRG1* was constructed, which was sourced from the pUCATPH plasmid and contains a hygromycin B resistance gene *hph* as a selectable marker. DNA of *T. longibrachiatum* SMF2 was isolated and used as a template to PCR amplify the upstream and downstream fragments of the *TRG1* gene using primer pairs LG1-1/LG1-2 and LG1-3/LG1-4 respectively, followed by primer pairs LG1-1P/LG1-2P and LG1-3P/LG1-4P, harboring appropriate restriction sites[19, 20]. The resulting fragments were recovered and purified for use as upstream and downstream recombination arms in the construction of the vector. All the primers used in the experiment were shown in Table 1.

First, the fragment *TRG1*-U, which was amplified using the primer pair LG1-1P/LG1-2P, and the vector pUCATPH was digested with Sph I and Hin dIII, and then ligated by T_4_ ligase. Then, another DNA fragment *TRG1*-D, which was amplified by the primer pair LG1-3P/LG1-4P, was digested with Kpn I and Sal I and ligated into the recombinant plasmid pUCATPH-U, which was digested using the same enzymes. The recombinant vector pUCATPH-*TRG1* was identified and subsequently used to transform *T. longibrachiatum* SMF2 protoplasts utilizing a restriction enzyme-mediated integration (REMI) transformation technique [19, 20]. Transformants were selected on hygromycin-containing medium. Positive transformants were initially verified by PCR analysis.Then Southern hybridization and RT-PCR were performed to confirm the single-copy integration of the *hph* cassette and the absence of *TRG1* gene in the knockout mutant Δ*TRG1*. All molecular experiments followed standard protocols [21].

### Phenotypic Characterization of the Mutant Δ*TRG1*

The wild-type strain (WT) and the mutant Δ*TRG1* were cultured on PDA medium for 7 days to assess growth rate, colony morphology, spore production, and other phenotypic characteristics. Assays were conducted in triplicate (n = 3). The colonies diameter of the WT and Δ*TRG1* were measured daily to evaluate their relative growth rate.


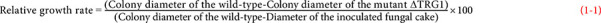


### Biological Control Assays of the Mutant Δ*TRG1*

*Botrytis cinerea* is an important plant pathogenic fungus, and *T. longibrachiatum* *SMF2* can be used to control various plant gray molds, so we select *Botrytis cinerea* to conduct biological control experiments. The confrontation culture method was used to determine the inhibitory effect of the WT strain and mutant Δ*TRG1* on the growth of the fungus *B. cinerea* T4. The strain of the WT and mutant Δ*TRG1* were co-inoculated with *B. cinerea* on PDA medium. *B. cinerea* cultured alone was used as a negative control. Assays were conducted in triplicate (n = 3). The diameter of *B. cinerea* colonies were measured on day 6 after inoculation in the different treatment groups and the inhibition rate was calculated using the following formula:


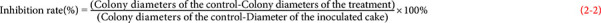


Detached rose leaves were used to evaluate the protective efficacy of the mutants against rose gray mold disease caused by *B. cinerea*. Firstly, the detached leaves were inoculated with a mycelial cake of *B. cinerea* T_4_, and placed on the filter paper spread in the culture dish, maintaining humidity with sterile water. Two days post-inoculation, the spore suspensions (1 × 10^6^ spores/mL) of the WT and mutant Δ*TRG1* were sprayed on the leaves using sterile syringes. The control group received an equivalent volume of sterile water sprayed onto the pathogen-inoculated leaves. Assays were conducted in triplicate (n = 3). Subsequently, the disease severity of leaves was assessed on day 7 after spraying with spore suspensions of the WT andΔ*TRG1*, the disease index and protective effect were calculated according to formulas 2-3 and 2-4 respectively, to determine the control effect of the WT andΔ*TRG1* on rose gray mold disease [11]. (Disease grading criteria: 0: No symptoms;1: Lesion area <12.5% of leaf area; 2: 1.5% of leaf area ≤ Lesion area <25% of leaf area; 3: 25% of leaf area ≤ Lesion area <50% of leaf area; 4: Lesion area ≥50% of leaf area) [11].


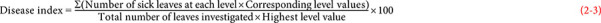



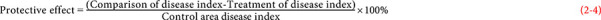


### Antifungal Assay of the Cell-Free Fermentation Supernatant

After culturing on PDA medium for 5 days, the spores of the WT strain and the mutant Δ*TRG1* were collected and spore suspension prepared was prepared (1 × 10^8^ spores/mL). This suspension was inoculated into PDB medium and cultured in a shaking incubator, at 150 rpm, 25°C for five days. The culture mixture was filtered through four layers of gauze to remove hyphae, and the filtrate was centrifuged for 10 min, at 12,000 rpm. The resulting supernatant was filtered through a 0.22 μm sterile filter membrane. This cell-free supernatant was then mixed with sterile PDA in a ratio of 1:10 (v/v), mixed evenly, and poured into Petri plates. *B. cinerea* T_4_ was inoculated onto the plate and cultured at 25°C for 4 days. The diameter of the colony was measured, and the inhibition rate was calculated according to Formula 2-2.

### Detection of Induced Resistance-Related Enzymes

The fermentation broth of the WT and mutant Δ*TRG1* was prepared as described in Section 2.6. This broth was sprayed onto detached rose leaves, with sterile water was used as a comparative control. Assays were conducted in triplicate (n = 3). The leaves were sampled on the 1st, 3rd, and 5th days post-treatment to measure the content of soluble protein and the activities of key resistance-related enzymes, such as superoxide dismutase (SOD) and catalase (CAT). Enzyme activities were measured following the methods described in Güneş [22].

### High Performance Liquid Chromatography(HPLC) Analysis

The WT and Δ*TRG1* strains were cultured on PDA plates for 7 d, spores were collected, inoculated into solid fermentation medium (9 g dry wheat bran with 45% moisture content, Grass powder 1g, KH_2_PO_4_ 0.048 g, (NH_4_)_2_SO_4_ 0.048 g, CaCl_2_ 0.02 g, MgSO_4_·7H_2_O 0.02 g) for 7 d, at 28°C. The fermented material was extracted with 100 mL anhydrous ethanol for 4 h. After filtration and centrifugation at 12,000 rpm, the supernatant was concentrated and dried using a rotary evaporator. The dried extract was resolubilized in 10 ml methanol and filtered through a 0.22 μm filter. Then take 10 μL of the solution for HPLC analysis[23], The HPLC parameters were set as follows: mobile phase was methanol and water(81:19); flow rate was 1 mL/min. High-performance liquid chromatography (HPLC), equipped with an ultrasonic degasser (DGU-20A5R), a binary high-pressure gradient pump (LC-30AD), an automatic sampler (SIL-30AC), a column oven (CTO-20AC), a photodiode array detector (SPD-M30A), and a communications bus module (CBM-20A). The column specifications were 250 × 4.6 mm in size, with a 5 μm C18 packing material. And the detection wavelength of HPLC is 254 nm.

### Statistical Analysis

The experimental data were analyzed by ANOVA using IBM SPSS Statistics 26 and the means of different treatments were compared by using Least Significant Difference (LSD) method. Figures were plotted using OriginPro 2023 [24].

## Results

### The Transcription Regulator Gene *TRG1* Is Located on the NRPS Gene Cluster Containing the NRPS Gene *tlx1*

**Fig. 1:**
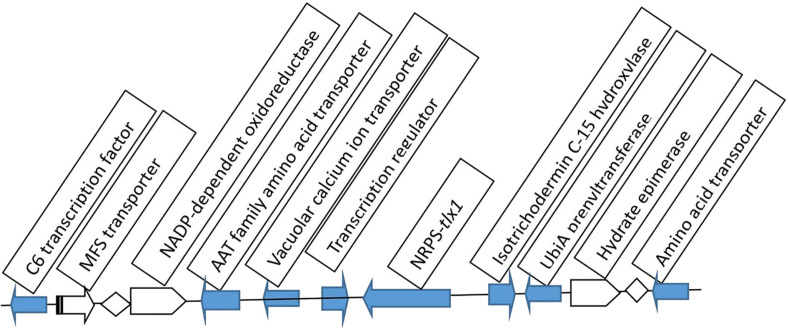
The location of the *TRG1* gene within the non-ribosomal peptide synthetase (NRPS) gene cluster containing *tlx1*.

We investigated the gene cluster where the NRPS gene *tlx1* is located, and found that the predicted gene cluster contains genes that are likely related to peptide modification, transport, and synthesis regulation (Fig. 1). In particular, a predicted C6 transcription factor gene (KB290*LG1*_475) and a transcription regulator gene (*TRG1*) were found, both of which were associated with gene expression regulation (Fig. 1). In particular, the C-terminus of the predicted protein of the gene *TRG1*, which is closest to the NRPS gene *tlx1* on the gene cluste possesses the conserved domain characteristics of the TRG2 regulatory protein.

### Construction and Identification of *TRG1* Mutant in *T. longibrachiatum* SMF2

**Fig. 2:**
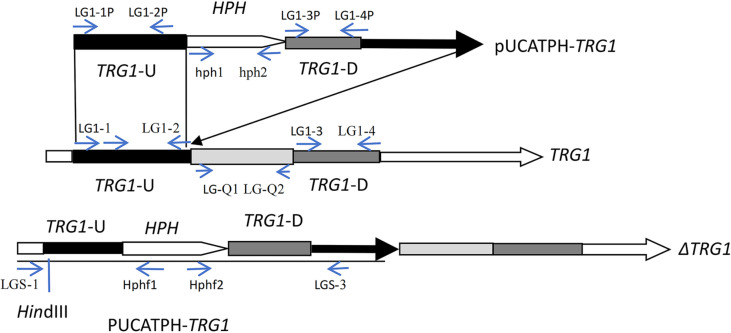
Schematic diagram illustrating theΔ*TRG1* knockout strategy, showing the recombinant vector (pUCATPH-*TRG1*) and the predicted homologous recombination event. pUCATPH-*TRG1*: The knockout vector; *TRG1*: The *TRG1* gene; Δ*TRG1*: Diagrammatic representation of recombinant gene (The figure contains the names and positions of the gene fragments, primers, and enzyme cutting sites).

The upstream and downstream recombination arms of *TRG1* were amplified by PCR with primer pairs LG1-1P/LG1-2P and LG1-3P/LG1-4P, yielding products of 750 bp and 1000 bp, respectively. These two fragments were inserted into the recombinant plasmid pUCATPH-*TRG1* using a two-step method, with the two inserts surrounding the hygromycin resistance gene *HPH*. A schematic of the construction of the recombinant vector pUCATPH-*TRG1* is presented in Fig. 2.

**Fig. 3:**
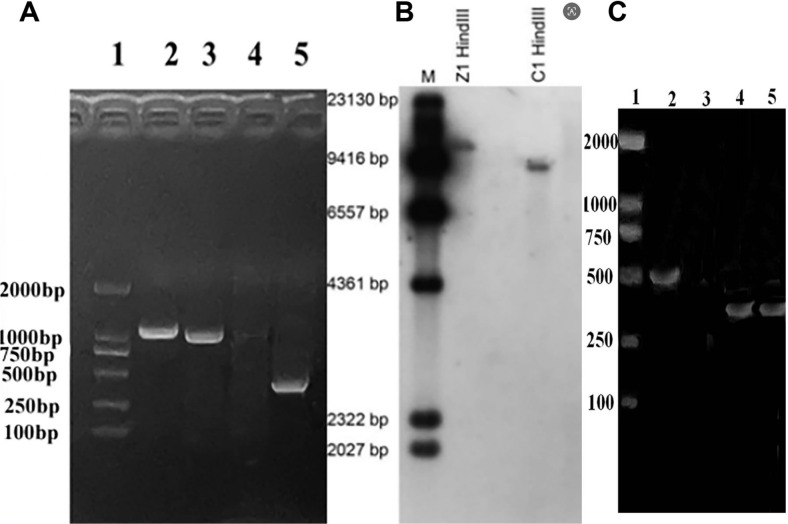
Validation of the gene recombinant in Δ*TRG1*. (**A**) PCR analysis of the gene deletion in the mutant Δ*TRG1*. (Lane 1: Size Markers; 2: PCR amplification product using primer pair *hph*1/*hph*2; 3: PCR amplification product using the upstream recombination primers; 4: Absence of PCR product of using the downstream recombination primers; 5: PCR amplification of the missing fragment). (**B**) Southern blotting verification of vector insertion and gene recombination in Δ*TRG1* mutant (Lane 1: Size Markers; 2. Z1:Hybridization product using Mutant Δ*TRG1* DNA digested with HindIII as the template; 3. C1: Hybridization product using the WT DNA digested with Hind? as the template). (**C**) RT-PCR product using *TRG1* primer set on cDNA derived from WT and Δ*TRG1* strains (Lane 1: Size Markers; 2: WT; 3: Mutant Δ*TRG1*;4:WT of*β-tubulin*; 5: Mutant of*β-tubulin*)

A total of 78 transformants obtained through REMI transformation were screened. After culturing for multiple generations to ensure stable inheritance of hygromycin resistance, a recombinant mutant was identified, designated as Δ*TRG1*, was identified via PCR and hybridization screening. Initial PCR and sequencing analysis confirmed the correct upstream recombination, yielding a product that contained the genomic fragment upstream of the recombination site, the upstream recombination arm, and a partial fragment of the HPH gene (Table 2). Meanwhile, the PCR results showed that the missing gene fragments of *TRG1* on the vector could be obtained from the mutant DNA (Fig. 3A). Further validation was performed by Southern blotting. When hybridization was performed with the upstream recombination arm as a probe, only one hybridization band was obtained in both the WT and mutant Δ*TRG1*(Fig.3B), indicating a successful homologous recombination in the mutant Δ*TRG1*. If the recombinant vector was a heterologous insertion without gene recombination event, there will be two hybridization bands, one with the same as the WT (Shorter than 9,000 bp), and the other one will be longer than the recombinant vector (Figs. 2, Fig. 3A and 3B). In short, the vector pUCATPH-*TRG1* was introduced into the *T. longibrachiatum* SMF2 genome via a single crossover event, resulting in the disruption of the integrity gene, without any gene fragment deletion or heterologous insertion events in the genome. A diagram of the presumed recombination model is shown in Fig. 2, and the detected recombinant sequence is shown in Table 2.

Total RNA was extracted from both WT and mutant Δ*TRG1*, reversed transcribed, and the resulting cDNA was used as a template for PCR amplification of the *TRG1* gene and the positive control (*β-tubulin*) using the primer pairs LG-Q1/LG-Q2 and TUB1/TUB2, respectively. The results confirmed that the *TRG1* gene was normally transcribed in the WT strain, but *TRG1* transcription could not be amplified from the Δ*TRG1* mutant (Fig. 3C).

### The Mutant Δ*TRG1* Showed No Significant Difference in Morphology, Growth Rate and Spore Production Compared to the WT

**Fig. 4:**
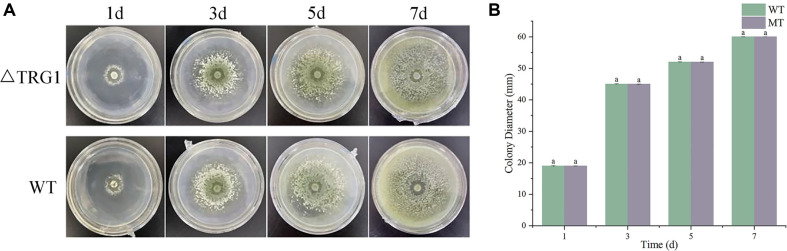
Comparison of growth rates between mutant Δ*TRG1* and the wild-type. Visual observations of colony growth over 7 days of culture (WT: *T. longibrachiatum* SMF2; Δ*TRG1*:*TRG1* knockout mutant). (**A**) Measurement of colony diameter of the WT and Δ*TRG1*. Data represent the mean ± sd. (n = 3). Different letters above the bars indicate a significant difference between the two strains within each timepoint as determined by an LSD test (*p* < 0.05).

The phenotypic characteristics of *T. longibrachiatum* SMF2 (WT) and the mutant Δ*TRG1* on PDA medium were observed and compared. Observation, colony diameter measurement, and calculation of the relative growth rate showed no significant difference in growth ability between the two strains within 7 days of culture (Fig. 4).

**Fig. 5:**
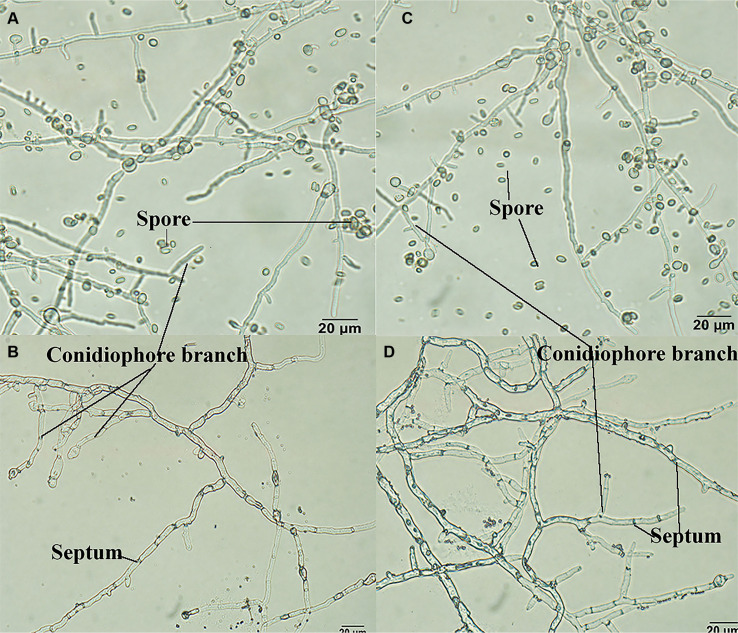
Comparison of hyphae and spores between mutant Δ*TRG1* and the wild-type. A/B: mutant Δ*TRG1*; C/D: the wild-type.

Microscopic observation of the hyphae and spores revealed that both strains began to produce septa three days after spore germination (Fig. 5). Both exhibited conidiophores branching straight to both sides, with spores single or clustered at the top. While the conidiophores of the mutants were generally shorter than those of the wild type (Fig. 5), this was a subtle difference.

Preparation of spore suspensions and hemocytometer counting showed that both Δ*TRG1* and WT spores were oval or nearly elliptical.The spore size of WT was (2.75~4.21) μm × (5.60~6.92) μm (width by length), while the spore size of the Δ*TRG1* was (2.67~4.05) μm × (4.43~5.95) μm, showing no significant change in spore size or yield (Fig. 5). These results indicate that the disruption of the *TRG1* gene does not significantly affect the primary morphological or reproductive characters of the fungus (Fig. 5).

### The mutant Δ*TRG1* showed a significantly lower inhibitory effect on *Botrytis cinerea*

**Fig. 6:**
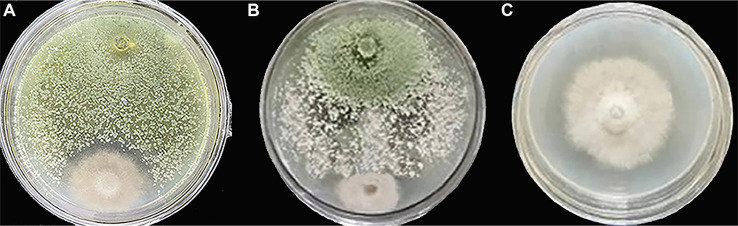
*In vitro* inhibition of *Botrytis cinerea* by WT and mutant Δ*TRG1* strains when co-cultured on PDA. (**A**) Δ*TRG1* and *B. cinerea*; (**B**) WT and *B. cinerea*; C: *B. cinerea*.

Although the mutantΔ*TRG1* showed no significant difference in morphology, growth rate and spore production compared to the WT, the inhibition activity of the mutant Δ*TRG1* were significantly lower than that of *T. longibrachiatum* SMF2 (WT) (Fig. 6). Both strains were able to inhibit the growth of *B. cinerea* over the 4-day culture period. On the fourth day, the inhibition rates were 71% for the WT and 60% for the mutant Δ*TRG1* (Table 3), with the WT strain demonstrating a significantly higher inhibition rate than the mutant Δ*TRG1* (LSD test, (*p* < 0.05).

### The control effect of mutant Δ*TRG1* on *Botrytis cinerea* was reduced on detached rose leaves

**Fig. 7:**
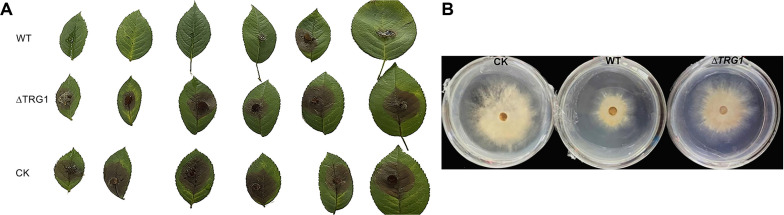
The control efficacy of the *TRG1* knockout mutant Δ*TRG1* against gray mold disease and its inhibitory effect on *Botrytis cinerea*. (**A**) The protective effect of the fermentation broth of Δ*TRG1* mutant and the WT strains on the gray mold disease of detached rose leaves. (**B**) *In vitro* inhibitory effect of the fermentation broth of the Δ*TRG1* mutant and WT strains on *B. cinerea* (CK:The Control; WT: *T. longibrachiatum* SMF2; Δ*TRG1*: *TRG1* knockout mutant).

After spraying the spore suspension of mutant Δ*TRG1* and the WT on Rose leaves, both strains alleviated gray mold symptoms compared with the control group. However, the WT strain showed a generally higher ability to decrease disease symptoms than that of mutant Δ*TRG1* (Fig. 7A, Table 4).

Seven days after treatment, the disease index of the leaves treated with the mutant Δ*TRG1* was 70.83, which was significantly higher than that of the WT strain (29.16). Correspondingly, the protective effect of Δ*TRG1* was 26.08%, which was significantly lower than the 69.8% observed for the WT strain (LSD test, (*p* < 0.05) (Table 4).

### The Inhibitory Effect of the Fermentation Broth of the Mutant Δ*TRG1* on *B. cinerea* Was Lower Than That of the WT

The cell free fermentation broths from both WT and Δ*TRG1* strains showed certain inhibitory effects on *B. cinerea*. However, the inhibitory effect of the Δ*TRG1* mutant broth was significantly lower than that of the WT strain, with an inhibition rate of 29% for the mutant Δ*TRG1* and 60% for the WT on the 4th day after inoculation (Fig. 7B, Table 5). This result strongly suggests that the reduced biocontrol ability of Δ*TRG1* is due to a reduction in secreted, active secondary metabolites.

### After the Treatment of the Mutant Δ*TRG1* Fermentation Broth, the Activity Level of the Resistance-Related Enzymes in the Detached Rose Leaves Was Lower Than That of the WT

Previous studies indicate that Peptaibols is involved in inducing plant disease resistance [9, 11] therefore, we examined the changes in plant soluble protein and resistance-related enzyme produced by the mutant fermentation broth.

The effect of the spore-free fermentation broths on the induction of systemic resistance in rose leaves was assessed by measuring soluble protein, superoxide dismutase (SOD), and catalase (CAT) activities.

**Fig. 8:**
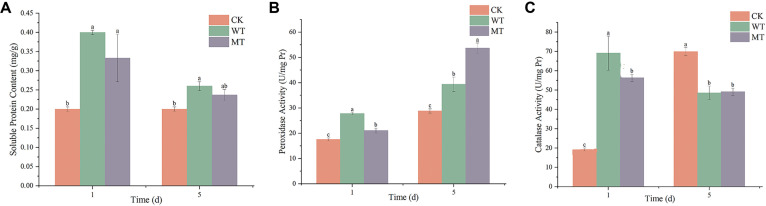
The activity level of the resistance-related enzymes indetached rose leaves after treatment with WT and Δ*TRG1* mutant fermentation broth. (**A**) soluble protein; (**B**) SOD activity; (**C**) CAT activity.

One day after treatment, the content of soluble protein was significantly higher in both treatment groups than in the control, with the WT treatment resulting in higher content than the Δ*TRG1* mutant. From the third day onward, the soluble protein of both treatments began to decrease (Fig. 8).

Similarly, the activities of SOD and CAT in rose leaves were higher than those of the control. Notably, the activities of both SOD and CAT in leaves treated with the WT fermentation broth were significantly higher than those treated with the mutant Δ*TRG1* on the 1st day (Fig. 8).

### HPLC Results Showed That the Yield of Peptaibols Produced by the Δ*TRG1* Was Significantly Reduced

**Fig. 9:**
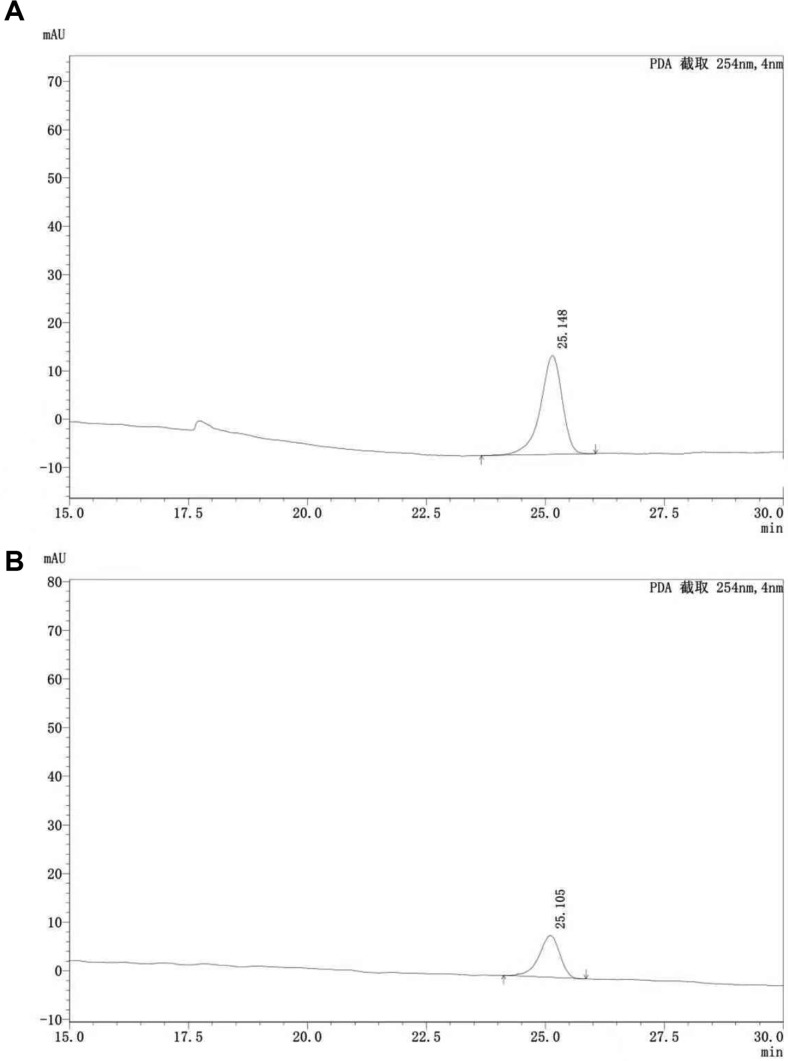
Comprise of the yield of peptaibols produced by the WT and mutant Δ*TRG1*. (**A**) The WT; (**B**) mutant Δ*TRG1*.

Analysis of the fermentation extracts by HPLC provided direct evidence of metabolite reduction (Fig. 9). Although both the wild-type strains and mutant Δ*TRG1* showed peptaibol peaks at 25 min, which corresponds to Trichokonin VI, the main active peptide of *T. longibrachiatum*, the difference in peak area size was significant. Peak area calculations showed that the area of *T. longibrachiatum* SMF2 (WT) and the mutant were 653190 and 2603686 respectively, which indicating Trichokonin VI yield of wild type was 2.5 times higher than that of the mutant Δ*TRG1* (Fig. 9). This result directly confirms that the transcription regulator gene *TRG1* is essential for the high-yield production of peptaibols, explaining the observed reduction in all functional biocontrol assays.

## Discussion

*Trichoderma* spp. are important biocontrol agents, effective against numerous pathogens including fungi (*Fusarium oxysporum*, *B. cinerea*), oomycetes (*Phytophthora infestans*), and bacteria (*Erwinia*) [25, 26]. These fungi produce a variety of secondary metabolites that facilitate the inhibition of pathogenic microorganisms, promote plant growth, and enhance their own colonization [27, 29]. Among these metabolites, peptaibols, synthesized through NRPS pathways, are particularly important for microbial inhibition, plant growth promotion, and the induction of plant resistance [8]. Research has established that NRPS genes are massive, with some, like *tex1*, encoding proteins exceeding 20,000 amino acid residues [30]. Targeted disruption of these core core NRPS genes, such as *tex1* or *tex2* leads to the loss or partial loss of peptaibol production [30, 31].

Furthermore, the genes involved in peptaibol biosynthesis, processing, and transport often form NRPS gene clusters that include associated regulatory genes [32]. For instance, in *T. longibrachiatum* SMF2, two NRPS genes, *tlx1* and tlx2, responsible for the production of the 20-amino acid Trichokonin VI (TKA) and 12-amino acid TKB, have been identified [30, 31]. Known regulatory elements, such as the glucose sensor homolog TlSTP1 and the LaeA homolog TlLAE1, have been shown to play important roles in regulating peptaibol production in this species [33]. Despite this progress, many questions regarding the precise transcriptional regulatory mechanisms remain unresolved.

In this study, bioinformatics analysis confirmed that the predicted gene cluster containing the NRPS gene *tlx1* included genes that may be involved in peptide modification, transport, and synthesis regulation. We focused on the transcription regulator gene *TRG1* by constructing a knockout mutant (Δ*TRG1*). Phenotypic characterization showed that the Δ*TRG1* mutant exhibited no significant alterations in growth rate, colony morphology, spore production, and spore size compared to the WT strain, indicating *TRG1* may not be essential for primary metabolism. However, functionally, the Δ*TRG1* mutant exhibited lower in vitro inhibition activity against *B. cinerea* and poorer in vivo control effect on gray mold compared. Moreover, the Δ*TRG1* cell-free fermentation broth was less effective at inducing resistance-related enzymes (SOD and CAT) in rose leaves. In particular, the HPLC analysis of the fermentation broth showed that the yield of peptaibols produced by the mutant was only 40% of the WT yield (a 2.5-fold reduction). The synthesis of these anti-fungal and resistance-inducing metabolites is mediated by NRPSs, leading us to conclude that the transcriptional regulator gene *TRG1* is intimately involved in the positive regulation of peptaibol synthesis. The genes involved in fungal secondary metabolism generally exist in gene clusters regulated by complex, multi-level networks, with transcriptional regulation being the most critical [34]. Transcriptional regulation in filamentous fungi often involves two classes of transcription factors (TFs): global TFs, which respond to external stimuli and regulate multiple biosynthetic gene clusters (BGC), and pathway-specific TFs, which mediate highly selective regulation, usually affecting only the BGC where they are located [35, 36].

The transcriptional regulator *TRG1* obtained in our study is located directly on the NRPS gene cluster, suggesting it belongs to a pathway-specific positive transcription factor. The phenotype of the Δ*TRG1* mutant showed a 60% reduction in peptaibol yield, strongly supporting this classification. This suggests that *TRG1* acts as a powerful enhancer of transcription for relevant genes within the *tlx1* cluster, potentially by binding to promoter regions. However, since the peptaibol production did not completely cease, it implies that *TRG1* is not an indispensable primary activator but rather a key positive modulator, suggesting that a basal level of expression or regulation by other factors remains.

Identifying transcriptional regulatory proteins like *TRG1* is essential for understanding the molecular mechanisms underlying the synthesis of these valuable secondary metabolites. This knowledge is paramount for promoting target product yield and preparing highly efficient biocontrol strains through targeted genetic engineering. To fully elucidate the regulatory network, our next steps will involve further investigation: (1) Comparative transcriptome analysis and creation of double mutants to identify which global transcription factors regulate *TRG1* expression; (2) Yeast one-hybrid assay to precisely locate the cis-elements bound by *TRG1* on cluster promoters; and (3) Yeast two-hybrid analysis to explore potential interacting proteins. This comprehensive molecular approach will clarify the mechanism of *TRG1* regulating peptaibols synthesis, providing the necessary molecular targets for rational bioengineering strategies to significantly improve peptaibol yields for agricultural applications.

## Figures and Tables

**Table 1:** List of primers used in the experiment.

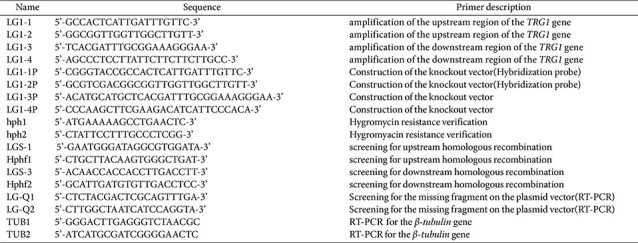

**Table 2:** The detected recombinant sequence.

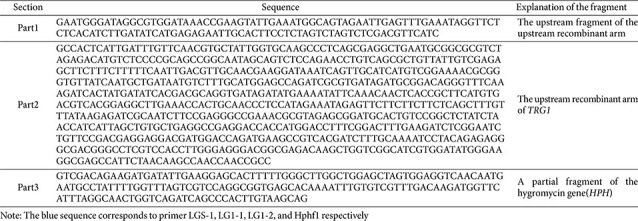

**Table 3:** Inhibition rate of the WT and mutant Δ*TRG1* on *Botrytis cinerea*.

Treatment	Colony diameters (mm)	Inhibition rate (%)
CK	78.21±0.23 a	-
*T.longibrachiatum* SMF2	26.33±0.51 c	71
Δ*TRG1*	34.67±0.44 b	60

Note: Data represent the mean ± sd. (n = 3); CK:blank control; c,b: Represents a significant difference relative to a, by an LSD test (*p* < 0.05), and a significant difference between the two treatments.

**Table 4:** The control effect of the WT and mutant Δ*TRG1* against rose gray mold disease of detached leaves and petals.

Treatment	Leaves Disease index	Leaves protective effect	PetalsDisease index	Petals protective effect
CK	95.83±2.12 a	-	91.25±1.98 a	-
*T.Longibrachiatum* SMF2	29.16±1.63 c	69.8±3.49 a	25.02±0.87 c	72.58±3.49 a
Δ*TRG1*	70.83±1.86 b	26.08±2.58 b	34.37±1.79 b	62.33±2.58 b

Note: Data represent the mean ± sd. (n = 3); CK:blank control; c,b: Represents a significant difference relative to a, by an LSD test (*p* < 0.05), and a significant difference between the two treatments.

**Table 5:** Inhibition rate of the WT and mutant Δ*TRG1* fermentation broth on *Botrytis cinerea*.

Treatment	Colony diameters (mm)	Inhibition rate (%)
CK	78.21±0.23 a	-
*T. Longibrachiatum* SMF2	34.6±0.42 c	60
Δ*TRG1*	57.16±0.21 b	29

Note: Data represent the mean ± sd. (n = 3). CK:blank control; c,b: Represents a significant difference relative to a, by an LSD test (*p* < 0.05), and a significant difference between the two treatments.
